# Evolutionary and environmental drivers of dry‐season deciduousness in a legume genus

**DOI:** 10.1111/nph.71148

**Published:** 2026-04-11

**Authors:** Cibele Cássia‐Silva, Jeannine Cavender‐Bares, Marcelo F. Simon, Leonel Herrera‐Alsina, Vinicius Marcilio‐Silva, Edeline Gagnon, Rafael Silva Oliveira, Jesús N. Pinto‐Ledezma

**Affiliations:** ^1^ Department of Ecology, Evolution and Behavior University of Minnesota Saint Paul MN 55108 USA; ^2^ Departamento de Biologia Vegetal Instituto de Biologia, Universidade Estadual de Campinas (UNICAMP) Campinas SP 13083‐862 Brazil; ^3^ Department of Organismic and Evolutionary Biology Harvard University Cambridge MA 02138 USA; ^4^ Embrapa Recursos Genéticos e Biotecnologia Brasília DF 70770‐901 Brazil; ^5^ School of Mathematical & Statistical Sciences, University of Galway Galway H91 TK33 Ireland; ^6^ School of Biological Sciences, University of Aberdeen 23 St Machar Drive Aberdeen AB24 3UU UK; ^7^ School of Natural Resource Sciences, North Dakota State University Fargo ND 58102 USA; ^8^ Department of Integrative Biology University of Guelph 50 Stone Road East Guelph ON N1G 2W1 Canada

**Keywords:** climate change, drought‐related strategies, *Mimosa*, phenology, plant diversification, seasonally dry tropical ecosystems

## Abstract

- Leaf deciduousness is a key drought‐avoidance strategy in tropical flora, reducing water loss during seasonal dry periods. While winter‐deciduousness in temperate regions is well‐understood, the evolutionary and environmental drivers of dry‐season deciduousness remain poorly explored.
- Using the genus *Mimosa,* a species‐rich and morphologically diverse lineage, we applied an eco‐evolutionary framework to investigate the role of dry‐season deciduousness across time and space. We combined a time‐sliced trait‐dependent diversification model, analyses of joint evolution of environmental niches in relation to leaf habit (deciduous vs evergreen), and phylogenetic multilevel models to test whether deciduousness influenced diversification and to identify its environmental drivers.
- Lineages switch from evergreen to deciduous habit more frequently before *c.* 7 Ma, whereas deciduous to evergreen transitions and increased speciation rates of evergreen lineages became dominant after this time. Deciduous lineages exhibited faster evolutionary rates along gradients of vapor pressure deficit, which also emerged as the strongest environmental predictor of deciduousness. However, most variation in leaf habit was explained by species‐level (nonphylogenetic) variation and phylogeny.
- Although deciduous lineages respond rapidly to atmospheric dryness, dry‐season deciduousness appears to be primarily shaped by the interplay between species identity and shared ancestry rather than by environmental conditions alone.

## Introduction

Leaf deciduousness contributes significantly to ecosystem functioning by maintaining living carbon stocks and sustaining productivity during unfavorable conditions, such as seasonal drought and freezing temperatures (Chabot & Hicks, 1982; Givnish, 2002). In tropical angiosperms, deciduousness likely originated in tropical and warm‐subtropical regions with mild winter atmospheric dryness, evolving primarily as an adaptation to predictable seasonal drought or water deficits (Axelrod, 1966), referred to as dry‐season deciduousness (De Souza *et al*., 2020; Anujan *et al*., 2026). By contrast, in high‐altitude angiosperms, winter‐deciduousness may have arisen as a preadaptation or exaptation – traits that evolved before new selective pressures but later conferred advantages (Gould & Vrba, 1982) – facilitating their subsequent expansion into frost‐prone temperate regions (Axelrod, 1966). Alternatively, winter‐deciduousness may have evolved *in situ* after woody evergreen angiosperms were exposed to freezing climates (Zanne *et al*., 2014), or it may have arisen in parallel with a gradually cooling climate (Edwards *et al*., 2017).

In tropical flora, dry‐season deciduousness is a key drought‐avoidance strategy that minimizes water loss by reducing the transpiring surface area and conserving moisture in long‐lived tissues during the dry season (Chabot & Hicks, 1982; Oliveira *et al*., 2021). Differential responses of drought‐deciduous and evergreen species to variations in water availability have been documented across tropical ecosystems (Souza *et al*., 2015; Oliveira *et al*., 2021; Wright *et al*., 2021; Vargas *et al*., 2022). While deciduous species decouple themselves from atmospheric water demand during the dry season, evergreen species tend to exhibit higher embolism resistance as water availability decreases (Oliveira *et al*., 2021; Vargas *et al*., 2022). At a global scale, drought‐deciduous species are more commonly associated with drier and more seasonal environments than evergreen species across the tropics (Vargas *et al*., 2022). In seasonally dry tropical forests, deciduous species typically rely on shallow soil water and greater tissue water storage, regardless of wood density (Wright *et al*., 2021). These studies suggest that current environmental variables influence the distribution of deciduous clades; however, the impact of environmental factors over evolutionary timescales remains poorly understood, particularly when compared to winter‐deciduous lineages.

Leaf habit in tropical ecosystems varies along a continuum – from evergreen and semideciduous species, which lose only a portion of their leaves and thus maintain the soil–plant–atmosphere continuum during the dry season (their responses to dry‐season resembling those of evergreen species (Kunert *et al*., 2021)), to brevi‐deciduous (species experience brief periods of complete leaf loss, but this deciduous period does not necessarily coincide with the driest time of the year), and fully deciduous forms (Bowman & Prior, 2005) – all of which can co‐occur within the same biome or even at small geographical scales (Souza *et al*., 2015; Silva de Miranda *et al*., 2018; Vargas *et al*., 2021, 2022). This variation reflects the combined influence of intrinsic plant traits and, especially, the complex interactions among multiple environmental factors (Sobrado, 1991; Kikuzawa, 1995; Givnish, 2002; Kunert *et al*., 2021). Although deciduousness is considered an adaptive response to predictable seasonal drought in seasonally dry tropical ecosystems (Axelrod, 1966), dry‐season conditions alone may not fully explain the observed leaf habit variation, even when leaf habits are simplified into a binary classification of deciduous vs evergreen (Vico *et al*., 2017; Schwartz *et al*., 2020). Plant water availability can be described along three key axes: atmospheric dryness, soil water availability during the dry season, and precipitation in the growing season – each representing a fundamental predictor of leaf habit (Vico *et al*., 2017; Grossiord *et al*., 2020; Oliveira *et al*., 2021). While atmospheric dryness and soil water availability are associated with leaf habit and drought‐induced mortality (Grossiord *et al*., 2020), precipitation in the growing season may also shape leaf habit, especially when the benefits of leaf shedding outweigh the energetic costs of regrowth (Sobrado, 1991; Vico *et al*., 2017). Soil nutrient availability is another key axis influencing variation in leaf habit over space (Givnish, 2002; Bowman & Prior, 2005). Leaf lifespan is strongly linked to nutrient supply (Wright *et al*., 2004), which can help explain differences between deciduous and evergreen strategies, in both temperate and tropical regions (Sobrado, 1991; Givnish, 2002; Hobbie & Gough, 2002; Ishida *et al*., 2006).

Within the leaf economics spectrum, deciduous and evergreen species represent contrasting ends, with resource‐acquisitive vs resource‐conservative strategies, respectively (Hikosaka *et al*., 2025). Understanding how these environmental factors have shaped functional traits, such as deciduousness, over evolutionary time is crucial for explaining patterns of angiosperm distribution across space and time (Zanne *et al*., 2018), and for understanding species coexistence at the community level (De Bello *et al*., 2025). This is particularly relevant in seasonally dry ecosystems, in which clarifying the role of dry‐season deciduousness in lineage radiation may reveal an underappreciated axis of tropical plant diversification (Harenčár *et al*., 2022). As the dry season in tropical ecosystems is becoming longer and drier – and is predicted to continue intensifying (Fu *et al*., 2013; Murray‐Tortarolo *et al*., 2016; Xu *et al*., 2022), investigating these evolutionary dynamics – even within specific clades – is critical for predicting vegetation responses to climate change and guiding species selection for ecological restoration (Cavender‐Bares *et al*., 2016; Díaz *et al*., 2016).

Model clades, defined by comprehensive taxon sampling, biogeographic and life‐history knowledge backgrounds, offer exceptional opportunities to investigate patterns of morphological evolution and historical biogeography in plants (Cavender‐Bares, 2019; Donoghue & Edwards, 2019). Smaller, but well‐sampled clades have been especially valuable in advancing comparative biology by serving as focused systems for in‐depth, multidimensional studies (Donoghue & Edwards, 2019). Studies based on model clades enable a deeper understanding of the mechanisms underlying the evolution of key adaptive traits (Fontes *et al*., 2025), such as deciduousness (Edwards *et al*., 2017). This is particularly important when focusing on clades with limited variation in the trait of interest because it reduces ascertainment bias, which is the tendency in comparative studies to focus only on traits that are easily variable and measurable (Beaulieu & O'Meara, 2018). Dry‐season deciduousness is a conspicuous trait in tropical flora, yet it remains sparsely documented (Laughlin *et al*., 2023).

Among angiosperms, the Fabaceae family ranks as the third most species‐rich family and includes the genus *Mimosa*, a morphologically and ecologically diverse clade comprising trees, shrubs, and climbers distributed across rainforest, savanna, seasonally dry and temperate biomes (Särkinen *et al*., 2011; Simon *et al*., 2011). Although several *Mimosa* species, such as *Mimosa pigra* L., one of the world's most problematic invasive plants (Kato‐Noguchi, 2023), have been introduced to regions beyond their native range, including East Africa, Madagascar, the Indian subcontinent, and the Andaman Islands, according to the World Checklist of Vascular Plants (WCVP; Govaerts *et al*., 2021) dataset, *the genus* is primarily distributed across the tropical America (Simon *et al*., 2011). Within its native range, *Mimosa* species are ecologically prominent components of the understory in major lowland tropical biomes, particularly in seasonally dry ecosystems – those characterized by a pronounced dry season – such as savannas and seasonally dry forests/succulent biome (Särkinen *et al*., 2011; Simon *et al*., 2011; Dexter *et al*., 2015). Following Schrire *et al*. (2005), we use the term *succulent biome* to refer to seasonally dry forests, hereafter. The *Mimosa* genus has undergone remarkable radiation in fire‐prone and grass‐rich open habitats such as the South American savanna (Cerrado) (Simon & Proença, 2000; Simon *et al*., 2009). Within the succulent biome – characterized by small‐leaved, fire‐sensitive, and drought‐deciduous vegetation – *Mimosa*, along with other Fabaceae lineages, shows exceptional diversification and strong niche conservatism (Särkinen *et al*., 2011; DRYFLOR *et al*., 2016; Gagnon *et al*., 2019). The prevalence of drought‐avoidance traits in the succulent biome suggests that woody plants lacking evolutionary adaptations to dry season are unlikely to thrive (Pennington *et al*., 2009), raising important questions about the role of drought‐related traits in enabling *Mimosa* to radiate into that biome. Although fire‐adaptive traits have been examined in relation to *Mimosa* diversification within the fire‐prone Cerrado (Simon *et al*., 2009), traits that have potentially enabled understory clades to thrive in other seasonally dry ecosystems – such as the succulent biome – remain poorly explored (Särkinen *et al*., 2011; Simon *et al*., 2011; Harenčár *et al*., 2022).

**Fig. 1:**
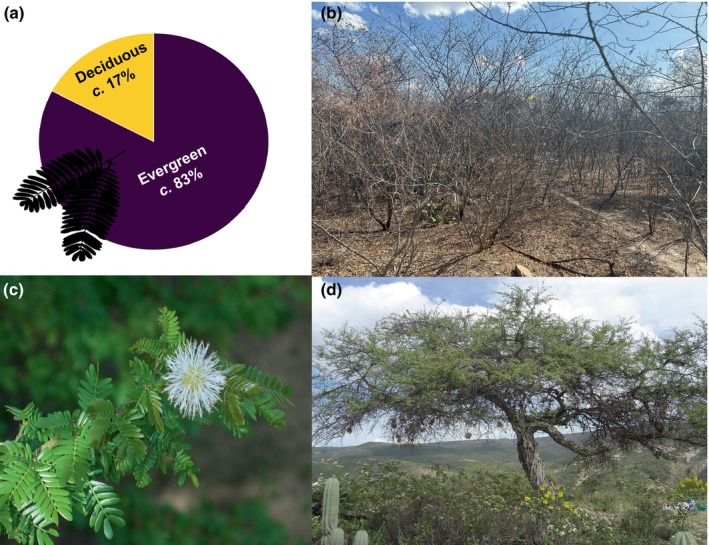
Leaf habit variation and morphological diversity in *Mimosa*. (a) Distribution of leaf habit types (deciduous vs evergreen) among the 278 *Mimosa* species with available leaf habit data included in the phylogeny. (b) The tree *Mimosa tenuiflora* (Willd.) Poir during the peak of the dry season in northeastern Brazil, within the succulent biome (Caatinga). (c) Flower diversity in *Mimosa hexandra Micheli*, a shrub or tree species primarily found in tropical rainforests (*Source*: World Checklist of Vascular Plants dataset; Govaerts *et al*., 2021). (d) The tree *Mimosa texana* (A.Gray) Small, which may also exhibit subshrub or shrub growth‐forms and occurs mainly in desert or succulent biomes (World Checklist of Vascular Plants dataset; Govaerts *et al*., 2021). Photo credit: Juliana Santos‐Silva (b) and Marcelo Simon (c, d). The *Mimosa* leaf silhouette in (a) was obtained from PhyloPic (http://phylopic.org) and is in the public domain.

Here, we leverage species occurrences, morphological, and phylogenetic data of the genus *Mimosa* (Fabaceae) to explore the relationship between the dry season and deciduous leaf habit, hypothesizing that deciduousness in this group functions as a drought‐avoidance strategy. Thus, we refer to it as drought‐deciduousness. *Mimosa* exhibits a comprehensive taxon sampling, well‐characterized biogeographic history (Simon *et al*., 2011), and limited occurrences of the deciduous leaf habit in species within the genus (Fig. 1), which contributes to reducing ascertainment bias (Beaulieu & O'Meara, 2018). At the same time, the deciduous leaf habit cannot be considered a rare trait in *Mimosa*, as it occurs in more than 10% of the sampled species (Fig. 1; Davis *et al*., 2013). These characteristics make *Mimosa* an excellent model for eco‐evolutionary studies (Folk *et al*., 2018; Donoghue & Edwards, 2019; Ringelberg *et al*., 2023). Using *Mimosa* as a model group, we investigated the influence of environmental regimes on the patterns of leaf habit variation (deciduous vs evergreen) and assessed the potential role of leaf deciduousness in facilitating the radiation of *Mimosa* into seasonally dry ecosystems and deserts across evolutionary time and geographic space.

First, we evaluated whether leaf habit variation (deciduous vs evergreen) influenced shifts in diversification rate regimes in *Mimosa* at two time points: 15 and 7 million years ago (Ma). This approach considers that the association between leaf habit and speciation rates may vary over time (Donoghue & Edwards, 2019; Herrera‐Alsina *et al*., 2019) and allows us to test whether dry‐season deciduousness is linked to the expansion of succulent biomes and tropical savannas, respectively, in the Americas. Fossil and phylogenetic evidence suggest that the succulent biome expanded, at least in parts of its current geographic range, such as South America, between *c*. 17 and 12 Ma (Burnham & Johnson, 2004; De Queiroz & Lavin, 2011), although an earlier origin between 30 and 20 Ma has also been proposed for the Mexican succulent biome based on a dated phylogeny (Becerra, 2005). Meanwhile, the geographic expansion of the tropical American savannas is estimated to have occurred no later than 10–4 Ma, based on both fossil records and molecular phylogenies (Osborne & Beerling, 2006; Simon *et al*., 2009). These time frames, 15 and 7 Ma, also coincide with the gradual diversification of major *Mimosa* lineages (Simon *et al*., 2011), providing a meaningful temporal context for exploring trait–diversification relationships in the genus. Second, we assessed whether variation in leaf habit (deciduous vs evergreen) was associated with differences in environmental niche evolution. We fitted several evolutionary models to a set of environmental variables characterizing the *Mimosa* environmental niche space. Specifically, we tested the role of leaf habit in influencing evolutionary rates, niche optima, and the strength of selection around environmental variables. Third, we evaluated which environmental factors are most strongly associated with dry‐season deciduousness across species, to better understand the ecological conditions linked to the occurrence of this drought‐avoidance trait.

## Materials and Methods

### Phylogenetic tree, leaf habit classification, and occurrence data

We used an ultrametric, time‐calibrated molecular phylogenetic tree of the genus *Mimosa* L. (Leguminosae) generated by Vasconcelos *et al*. (2020). This tree was inferred from 543 plastid molecular sequences and constructed under the GTR + I + G substitution model. Calibration points followed the approach proposed by Simon *et al*. (2011). For our analysis, we pruned outgroups and subspecies from the phylogenetic tree (Vasconcelos *et al*., 2020), using the packages ape (Paradis *et al*., 2004) and phytools (Revell, 2012) in R (R Core Team, 2025). This resulted in a tree with 336 tips, which was used in our analysis unless otherwise stated.

The classification of *Mimosa* species as deciduous or evergreen was based primarily on expert taxonomic assessments and revisions, supplemented by information from specialized datasets such as the WCVP (Govaerts *et al*., 2021). Although leaf habit in tropical trees exists along a continuum – including variation in the duration of the leafless period and the proportion of canopy shed (Givnish, 2002; Kunert *et al*., 2021), our survey suggests that such nuanced variation is less common among understory plants, which comprise the majority of *Mimosa* species. Most available data on *Mimosa* refer to whether a species is generally classified as deciduous – defined here as shedding all leaves during unfavorable conditions, such as the dry season or winter (Givnish, 2002; Bowman & Prior, 2005) – or evergreen. Species that do not completely shed their foliage were classified as evergreen, potentially including semideciduous species (those that partially lose their leaves). This binary classification is consistent with our hypothesis of deciduousness as a drought‐avoidance strategy in *Mimosa*, as semideciduous species tend to exhibit drought responses more similar to those of evergreens than to fully deciduous taxa (Bowman & Prior, 2005; Kunert *et al*., 2021). Of the 327 *Mimosa* species included in the phylogeny, we obtained leaf habit data for 278.

### Occurrence data and environmental data

To characterize the environmental niches of *Mimosa* species and identify potential environmental predictors of deciduousness (corresponding to our second and third aims), we compiled species occurrence records from multiple reliable sources. These included the Botanical Information and Ecology Network (BIEN; Maitner *et al*., 2018), the Latin American and Caribbean Seasonally Dry Tropical Forest Floristic Network (DRYFLOR *et al*., 2016), the Global Biodiversity Information Facility (GBIF; http://www.gbif.org/), and the NeoTropTree database (http://www.neotroptree.info/; Oliveira *et al*., 2021). All occurrence records were filtered and cleaned to retain only those within each species' native range. Introduced occurrences were excluded using the rwcvp R package (Brown *et al*., 2023), which draws on geographic range data from the WCVP (Govaerts *et al*., 2021). This filtering step was essential to remove invasive populations and ensure that the extracted environmental conditions accurately reflected natural species distributions. To avoid overestimating range size for species with limited occurrence data, that is species with less than five occurrence records (Meyer *et al*., 2017), we generated spatially thinned random points within native ranges. Species ranges were delineated using convex hulls around cleaned occurrence points, following the rwcvp filtering protocol (Brown *et al*., 2023). Although convex hulls may overestimate range size and fail to capture spatial discontinuities, particularly for species distributed along complex geographic features such as mountain chains, they provide reliable estimates of plant richness over geographic space (Meyer *et al*., 2017). This approach was thus chosen to maximize the environmental and spatial variation encompassed by species with few occurrence records, while maintaining consistency across taxa.

We extracted key environmental variables for each species' polygon to generate random points, including annual mean temperature (BIO1), temperature seasonality (BIO4), maximum temperature of the warmest month (BIO5), annual precipitation (BIO12), precipitation of the driest month (BIO14), and precipitation seasonality (BIO15) from WorldClim (Fick & Hijmans, 2017), as well as soil moisture (water content) layers from the SoilGrids database (Poggio *et al*., 2021; https://soilgrids.org/). For each clipped environmental area, we generated 20 random points (without replacement) using the dismo R package (Hijmans *et al*., 2024). To reduce spatial autocorrelation, all points (both observed and randomly generated) were further filtered using a spatial thinning procedure with a minimum distance of 1.5 km, implemented in the spthin R package (Aiello‐Lammens *et al*., 2015). This workflow yielded a total of 226 *Mimosa* species: 182 with more than five valid occurrence records and 44 represented by randomly generated points, all used for extracting environmental predictors.

To identify environmental predictors of leaf habit variation (deciduous vs evergreen) in *Mimosa*, we selected a set of climatic variables linked to seasonal water stress and soil variables (Table 1). In tropical ecosystems, both water availability and atmospheric demand are known to strongly influence patterns of leaf flushing and leaf shedding (Borchert, 1994; Borchert *et al*., 2002; Schwartz *et al*., 2020). Among the climatic predictors, we included the aridity index (AI = precipitation/potential evapotranspiration), total precipitation during the growing season precipitation (GSP), and vapor pressure deficit (VPD). These variables capture complementary dimensions of atmospheric evaporative demand and water limitation across the native ranges of *Mimosa* species. AI was extracted from the Global Aridity Index and Potential Evapotranspiration v.3 dataset (Zomer *et al*., 2022), while GSP and VPD were obtained from the CHELSA climate database (Karger *et al*., 2021) covering the period from 1979 to 2013. We also included soil texture variables as proxies for water‐holding capacity and nutrient availability (Table 1), since soil texture influences both resource types that are critical for plant growth and survival (Pereira e Silva *et al*., 2011; Costa *et al*., 2013). This is particularly relevant because drought‐deciduous species in succulent biomes may rely on shallow soil water during the dry season (Wright *et al*., 2021). In addition, the short leaf lifespans typical of deciduous species are often associated with environments that have higher soil nutrient availability (Hobbie & Gough, 2002; Wright *et al*., 2004). Specifically, we used clay and sand content at a depth of 5–15 cm (Table 1), obtained from the SoilGrids database (https://soilgrids.org/). All environmental layers were standardized to a spatial resolution of 30 arc seconds (*c*. 1 km), and values were extracted for each spatially filtered occurrence point using the R packages sp (Bivand, 2008), raster (Hijmans, 2022), and terra (Hijmans, 2020).

**Table 1:** List of environmental variables used to describe the environmental niche of *Mimosa* and to assess its association with deciduousness.

Code (Unit)	Environmental variable	Biological significance	Database
AI	Aridity index = Precipitation/Potential evapotranspiration	Indicates the macroclimatic water availability that affects water available for plant physiological processes	Global aridity index and potential evapotranspiration database v.3 (Zomer *et al*., 2022)
VPD	Vapor pressure deficit (VPD) is a measure of atmospheric dryness. It compares the amount of moisture in the air to the maximum amount the air can hold	Indicates water deficit during the dry season. The balance between rainfall and atmospheric demand (expressed as VPD) is a key predictor of ecological dynamics, including plant leaf habit and drought‐induced mortality (Grossiord *et al*., 2020)	CHELSA (Karger *et al*., 2021)
GSP	Precipitation sum accumulated on all days during the growing season	Represents water availability during the period of plant growth	CHELSEA (Karger *et al*., 2021)
STF (%)	Soil texture fraction: Clay content Sand content	Soil texture strongly affects the availability of both water and nutrients for plant growth and survival. Clay soils, with their smaller particle size and greater surface area, generally retain more water and nutrients than sandy soils (Pereira e Silva *et al*., 2011; Costa *et al*., 2013). Clay soils have higher Cation Exchange Capacity, reflecting their ability to adsorb and release essential nutrients such as K^+^, Mg^2+^, and Ca^2+^, which enhances fertility and reduces nutrient loss (Nesic *et al*., 2015). Because drought‐deciduous species depend on shallow soil water and also soil nutrient availability (Wright *et al*., 2004), we used clay and sand content as proxies for soil water‐holding capacity and nutrient availability	SoilGrids 2.0 (Poggio *et al*., 2021)

Each variable is followed by its biological relevance and supporting references.

We assessed multicollinearity among the selected environmental variables using the variance inflation factor (VIF) with a threshold of 5 (Dormann *et al*., 2013) in the usdm R package (Naimi, 2012). Although the AI exhibited a high VIF due to its strong correlation with GSP (*r* = 0.93), we retained both variables because our hypotheses focus on water availability as a key driver of plant physiological processes and leaf deciduousness. Moreover, AI and GSP capture complementary ecological dimensions of water limitation. AI reflects long‐term atmospheric water stress, especially under unfavorable dry‐season conditions, whereas GSP represents seasonal water availability during the period of active plant growth. These two aspects of water availability may impose different selective pressures on leaf habit, justifying their inclusion to evaluate their relative ecological relevance. Consistent with this ecological perspective, long‐term increases in aridity have been shown to drive shifts in species composition in tropical forests, favoring drought‐tolerant species, which are often deciduous (Feeley *et al*., 2011). However, we explicitly evaluate the impact of correlation between AI and GSP in modeling environmental predictors of deciduousness (will be discussed later). In addition, we used Bayesian Additive Regression Trees (BART) to explore nonlinear and nonparametric relationships between environmental predictors and deciduousness and to evaluate variable importance independently of parametric assumptions. Together, these complementary approaches allowed us to assess whether AI and GSP capture redundant or distinct dimensions of water availability relevant to leaf habit.

### Impact of leaf habit on Mimosa diversification rates

To evaluate whether leaf habit variation (deciduous vs evergreen) is associated with variation in diversification rates and whether these rates change over time (first aim), we used maximum likelihood State‐dependent Speciation and Extinction (SSE) models (Maddison *et al*., 2007; Herrera‐Alsina *et al*., 2019). SSE approaches simultaneously model trait evolution (i.e. trait state/character transitions, leaf habit in this case), speciation, and extinction rates along the branches of a phylogenetic tree. We compared models in which speciation rates depended on leaf habit (hereafter ‘dependent’ models) with null models in which diversification was independent of leaf habit, ‘independent’ or hidden‐state models (Beaulieu & O'Meara, 2016; Herrera‐Alsina *et al*., 2019). Because the dependence of diversification on leaf habit may have changed over time, we also implemented time‐stratified models that allowed different diversification regimes before and after the specified time points, by dividing the evolutionary history of *Mimosa* into early and late stages (SecSSE model; Herrera‐Alsina *et al*., 2025). We used 15 Ma as the time point to separate early from late stage, and repeated the analysis with a 7 Ma time point corresponding to the approximate expansions of the succulent biome and savannas in tropical America, respectively (Burnham & Johnson, 2004; Becerra, 2005; Simon *et al*., 2009; De Queiroz & Lavin, 2011). This also allows to assess temporal sensitivity in diversification–trait associations. Specifically, we tested four time‐structured scenarios:

1. Dependent early + independent late
2. Independent early + dependent late
3. Dependent early + dependent late
4. Independent early + independent late

In each case, we also tested symmetric (i.e. equal transition rates between evergreen and deciduous) and asymmetric trait transition models. Additionally, we allowed either time‐constant or time‐variable trait evolution rates (e.g. transitions early ≠ transitions late). The most parameter‐rich models allowed asymmetric and time‐variable transitions (e.g. evergreen to deciduous early ≠ deciduous to evergreen early ≠ evergreen to deciduous late ≠ deciduous to evergreen late). Model fit was assessed using Akaike Information Criterion (AIC), with complexity penalized accordingly. To minimize the risk of identifying only local optima, we ran two replicates per model with different starting points in the likelihood algorithm.

### Leaf habit and evolution of environmental niche

To assess whether variation in leaf habit (deciduous vs evergreen) was associated with differences in environmental niche evolution (second aim), we used the *hOUwie* framework (Boyko *et al*., 2023), implemented via the R package ouwie (Beaulieu *et al*., 2012) available at https://github.com/thej022214/OUwie/ and applied to 226 *Mimosa* species with available environmental data. We fitted nine alternative evolutionary models (Table 2), all based on extensions of Brownian Motion (BM) and Ornstein–Uhlenbeck (OU) processes applied to scaled environmental variables. Each *Mimosa* species was assigned to one of two selective regimes (deciduous or evergreen), assuming an all‐rates‐different (ARD) model for transitions between regimes. The models differ in how they parameterize evolutionary processes, allowing for variation in the evolutionary rate (σ^2^), selection strength (α), and trait optimum (θ). Model names reflect the parameters allowed to vary across regimes: ‘V’ indicates variation in σ^2^ (e.g. BMV, OUV), ‘A’ allows α to vary (e.g. OUA), and ‘M’ allows θ to differ between regimes (e.g. OUM). Models with multiple letters (e.g. OUMV, OUVA, and OUMVA) allow combinations of these parameters to vary. Model fit was evaluated using Akaike weights (AICw), calculated from the corrected AIC for small sample sizes (AICc; Burnham & Anderson, 2004).

**Table 2:** Environmental niche evolution models, as applied to environmental variables for deciduous and evergreen *Mimosa* species.

Model	Description	Explanation	Interpretation
BM1	Single‐rate BM model (σ^2^)	The environmental niche evolves stochastically at the same rate for deciduous and evergreen species	No association between leaf habit and environmental niche evolution
BMV	Multirate BM model (σ^2^)	The environmental niche evolves stochastically, but at different rates for deciduous vs evergreen species	Leaf habit is associated with differences in the evolutionary rate of environmental niche
OU1	Single‐optimum OU model (θ)	The environmental niche evolves toward a single optimum value, regardless of leaf habit	No association between leaf habit and environmental niche optimum
OUA	OU model with distinct selective strengths (α)	The environmental niche evolves toward the same optimum value, but with different selection strengths for deciduous and evergreen species	Leaf habit is associated with differences in selection strength (α)
OUV	OU model with distinct evolutionary rates (σ^2^)	The environmental niche evolves toward the same optimum value, but under different stochastic rates for deciduous and evergreen species	Leaf habit is associated with differences in the evolutionary rate (σ^2^) of environmental niche
OUM	OU model with distinct optima (θ)	The environmental niche evolves towards different optimum values for deciduous vs evergreen, but under the same rate and selection strength	Leaf habit is associated with differences in environmental niche optima (θ)
OUVA	OU model with distinct rates (σ^2^) and strengths (α)	The environmental niche evolves toward the same optimum, but both the evolutionary rate and selection strength differ between leaf habit regimes	Leaf habit is associated with differences in evolutionary rate (σ^2^) and selection strength (α)
OUMV	OU model with distinct optima (θ) and rates (σ^2^)	The environmental niche evolves toward different optima and under different rates for deciduous vs evergreen species	Leaf habit is associated with differences in environmental niche optima (θ) and evolutionary rates (σ^2^)
OUMVA	OU model with distinct optima (θ), rates (σ^2^), and strengths (α)	The environmental niche evolves toward different optima, and under different rates and selection strengths for each leaf habit regime	Full association: leaf habit affects environmental niche optimum (θ), evolutionary rate (σ^2^), and selection strength (α)

### Modeling of environmental predictors of deciduousness

To evaluate the environmental drivers of deciduousness across 226 *Mimosa* species with available environmental data (third aim), we fitted a baseline Bayesian phylogenetic multilevel model using the brms R package (Bürkner, 2017). The response variable, leaf habitat (deciduous or evergreen), was modeled as binary under a Bernoulli distribution. We included five environmental predictors as fixed effects: AI, VPD, GSP, and soil texture components (clay and sand content; Table 1). All predictors were mean‐centered and scaled by two standard deviations before model fitting using the arm R package (Gelman *et al*., 2024), ensuring comparable effect sizes across variables. To account for phylogenetic nonindependence, we included a species‐level random intercept informed by a phylogenetic correlation structure. This was derived from a variance–covariance matrix based on the *Mimosa* phylogeny, calculated using the ape R package (Paradis *et al*., 2004), standardized to a correlation matrix, and implemented using the ‘*gr*’ function in brms. We also included a nonphylogenetic species‐level random intercept to capture residual interspecific variation unexplained by phylogeny (Supporting Information Notes S1). Normal priors (mean = 0, SD = 2) were used for fixed effects and brms's default priors (i.e. Student's *t*‐distributions (df = 3, mean = 0, SD = 2.5)) for the intercept and random effects. Although these priors may be considered moderately informative, we verified their suitability through prior–posterior predictive checks (PPCs) and sensitivity analyses using broader (less informative) prior scales, which yielded consistent parameter estimates (Fig. S1). The model was run using cmdstanr as backend with four Markov Chain Monte Carlo (MCMC) sampling chains, each with 10 000 iterations each (including 2000 warm‐up), thinning every 10 steps, and used *adapt_delta* = 0.95 to improve sampling efficiency and reduce divergent transitions. Model convergence and sampling quality were assessed using *R̂* statistics, effective sample sizes (ESS), and visual inspection of trace plots. Following Gabry *et al*. (2019), we assessed the reliability of our model using PPCs. PPCs were used to evaluate how well the model reproduces the observed data and whether the posterior predictive distribution adequately captures key statistical properties of the response variable (Gabry *et al*., 2019). In addition, we evaluated the robustness of model estimates to prior specification through a prior sensitivity analysis (Depaoli *et al*., 2020; Kruschke, 2021). Specifically, we implemented the power‐scaling approach available in the priorsense R package (Kallioinen *et al*., 2024), which systematically varies the strength of the prior relative to the likelihood. Conceptually, this approach is equivalent to widening or narrowing the prior distribution (e.g. increasing or decreasing the standard deviation of a normal prior), thereby allowing us to assess how strongly posterior estimates depend on prior assumptions. Because power‐scaling effectively alters prior dispersion, we additionally examined models with broader priors to evaluate whether posterior estimates were sensitive to prior specification and to ensure that posterior inferences were not driven by overly restrictive priors.

We further assessed the impact of collinearity between the AI and GSP by evaluating their contributions to the model's explained variance, as reflected in marginal and conditional Bayesian *R* ^2^. Specifically, we compared the baseline Bayesian phylogenetic multilevel model (full model), which included both AI and GSP, with reduced models in which each variable was excluded in turn. This approach allowed us to evaluate whether retaining both predictors improved the variance explained by the model and to ensure that the inferred effects were not driven by collinearity between these two water‐availability variables.

**Fig. 2:**
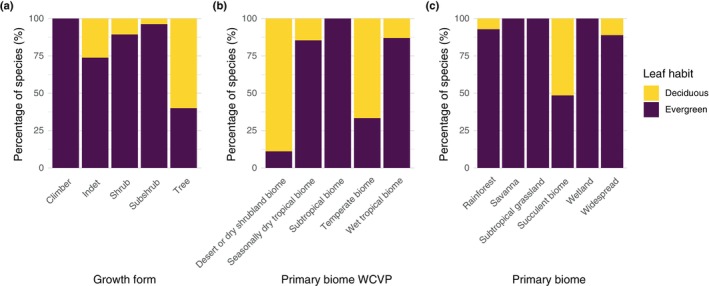
Proportion of deciduous and evergreen *Mimosa* species across growth‐forms and biome types, considering different biome classifications. (a) Proportion of deciduous and evergreen *Mimosa* species across different growth forms (climber, shrub, subshrub, and tree) based on the World Checklist of Vascular Plants (WCVP) dataset (Govaerts *et al*., 2021). *Indet*. indicates species with indeterminate or intermediate growth forms. (b) Proportion of deciduous and evergreen *Mimosa* species across major biome types, based on the WCVP dataset classification (Govaerts *et al*., 2021). (c) Proportion of deciduous and evergreen *Mimosa* species based on a finer‐scale biome classification, informed by expert knowledge and a literature review (Simon *et al*., 2009).

Given that growth‐form variation is an important axis of ecological strategy in *Mimosa*, with respect to not only climate other environmental pressures such as fire (Simon *et al*., 2009), and that deciduous *Mimosa* species are predominantly trees (Fig. 2a), we also compared the baseline Bayesian phylogenetic multilevel model with an alternative model including growth‐form as a fixed effect. In this formulation, growth‐form variation (Fig. 2a) was included as a fixed effect alongside environmental predictors. Note that before model fitting, growth‐form was also scaled by two standard deviations (Gelman *et al*., 2024). The baseline and growth‐form models differed only in the inclusion of growth‐form as an additional fixed effect, allowing us to evaluate whether growth‐form explains additional variation in leaf habit and increases the variance explained by the model, as reflected in marginal and conditional Bayesian *R* ^2^.

## Results

Deciduous *Mimosa* occur mainly in desert, dry shrubland, and temperate biomes (Fig. 2b), according to the broad biome classification of the WCVP (Govaerts *et al*., 2021). Under the finer‐scale biome classification derived from expert knowledge and literature (Simon *et al*., 2009), deciduous *Mimosa* are found primarily in succulent biomes, followed by widespread occurrence across more than three biomes and in rainforests (Fig. 2c).

### *Mimosa's* speciation is linked to leaf habit

We found that models assuming no temporal change in the dependence or independence of speciation rates on leaf habit performed poorly (Table S1). Models that incorporate a shift in diversification dynamics *c*. 7 Ma provided a better fit to the data than those assuming a shift at 15 Ma. Temporal models in which the *Mimosa* radiation began with a dependence of speciation rates on leaf habit also had little statistical support. Instead, the best‐supported model featured a dynamic in which speciation rates were initially independent of leaf habit but became dependent in the later stages of radiation, suggesting that leaf habit only became evolutionarily relevant in the last 7 Myr. Moreover, models allowing asymmetric transitions between leaf habits outperformed those with symmetric transition rates. We also found strong support for models in which transition rates vary through time (Table S1). Given the strong temporal component in the radiation of *Mimosa*, we describe below the two‐stage dynamics as inferred from the best‐performing model.

**Fig. 3:**
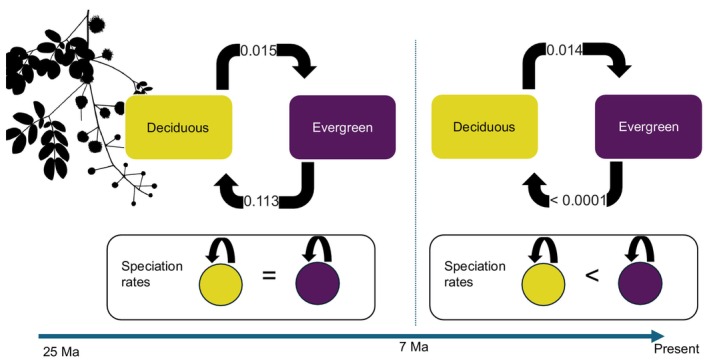
Temporal macroevolutionary dynamics of leaf habit in *Mimosa*, showing transition rates between deciduous and evergreen states and state‐dependent speciation rates over time. (Upper) Transition rates between leaf habits (deciduous vs evergreen); (lower) state‐dependent speciation rates. Models allowing a shift in diversification dynamics *c*. 7 million years ago (Ma) fit significantly better than those assuming a shift at 15 Ma. The best‐supported model indicates that transitions from deciduous to evergreen remained nearly constant over time (0.015 Ma^−1^ before 7 Ma, indicated by the vertical dashed line; 0.014 Ma^−1^ thereafter). By contrast, transitions from evergreen to deciduous leaf habit were markedly higher in the past (0.113 Ma^−1^ before 7 Ma) but nearly ceased in the last 7 Myr (< 0.0001 Ma^−1^). Speciation rates became associated with leaf habit only during the past 7 Ma, with evergreen lineages exhibiting higher speciation rates than deciduous lineages, whereas no state‐dependent differences were detected before 7 Ma. The silhouette represents *Mimosa lactiflua* Delile ex Benth., a deciduous species, and was derived from a photograph by Marcelo Simon.

During the early phase of *Mimosa* radiation, the transition rate from evergreen to deciduous leaf habit was considerably higher (0.113) than the reverse transition (*c*. 0.015; Fig. 3). At this stage, speciation rates did not differ between evergreen and deciduous species. However, in the late phase of radiation – within the last 7 Ma – evergreen species exhibited higher speciation rates than deciduous ones. While transition rates from deciduous to evergreen remained similar to the earlier period, rates from evergreen to deciduous declined to near zero (Fig. 3).

### Leaf habit influences key aspects of the environmental niches of *Mimosa*

**Fig. 4:**
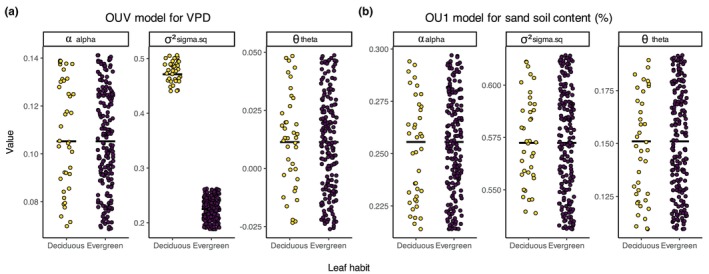
Evolutionary differences in environmental niche parameters (α: strength of selection; σ^2^: evolutionary rate; θ: trait optimum), between deciduous and evergreen *Mimosa* species. (a) For vapor pressure deficit (VPD), the best‐supported model was OUV (AICw = 0.95). The two regimes differed primarily in σ^2^, with higher values in deciduous species, indicating faster evolutionary rate responses in the VPD niche space. (b) For sand content (%), the best‐supported model was OU1 (AICw = 0.65), which assumes a single evolutionary regime. The lack of differences in α, σ^2^, and θ between leaf habit states suggests either environmental niche convergence or similar selective pressures acting on deciduous and evergreen species.

We found evidence for differences in environmental niche evolution between deciduous and evergreen lineages (Table S2). Our best‐supported models were OUV model for VPD (AIC_w_ = 0.95), and OU1 model for sand soil content (AIC_w_ = 0.65). Under the OUV model, deciduous lineages exhibited faster rates of VPD niche evolution than evergreen lineages (σ^2^_deciduous_ > σ^2^_evergreen_; Fig. 4a). This result suggests that evergreen species have a more conserved environmental niche with respect to VPD values over time. By contrast, the OU1 model for sand soil content indicated that all *Mimosa* lineages, regardless of leaf habit, are evolving toward a single environmental optimum (θ_deciduous_ = θ_evergreen_; Fig. 4b), suggesting shared niche constraints regarding sand content. In addition, we found less support for the best‐fitting models for AI (OUV; AICw = 0.40), precipitation in the growing season (OUA; AICw = 0.38), and clay soil content (OU1; AICw = 0.37).

### Deciduousness is explained by VPD and variation among species and lineages

The baseline Bayesian phylogenetic multilevel model showed good predictive performance based on PPCs (Fig. S2), and its results were qualitatively consistent across a range of reasonable prior scales, indicating that inference on environmental predictors of deciduousness was robust to prior choice (Table S3). Prior sensitivity analyses showed that widening the prior distributions increased coefficient magnitudes but did not alter the direction of effects or the overlap of credible intervals with zero (Table S3). Posterior distributions remained consistent across prior power‐scaling values, indicating that parameter estimates were robust to prior specification (Figs S3, S4).

**Fig. 5:**
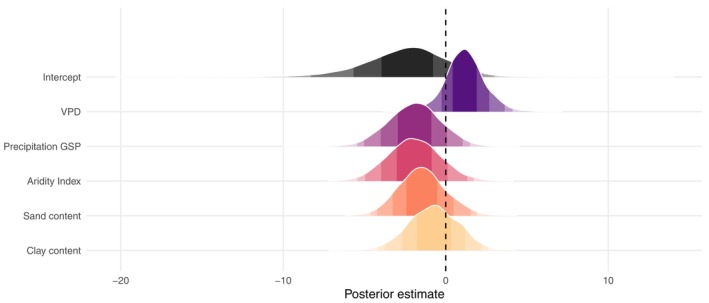
Posterior estimates of environmental predictors of leaf habit from a Bayesian phylogenetic multilevel model for *Mimosa*. Posterior distributions (colored densities) and 95% credible intervals for fixed effects include vapor pressure deficit, growing season precipitation, aridity index, and soil texture variables (sand and clay content, %). The vertical dashed line indicates a zero effect. Posterior estimates represent the direction and magnitude of each predictor's influence on the probability of a deciduous leaf habit. Positive values indicate increased probability of deciduousness, whereas negative values indicate increased probability of evergreen habit.

The baseline model explained a substantial proportion of variation in leaf habit among *Mimosa* species, with a conditional Bayesian *R* ^2^ of 0.80 (0.64: 0.94, 95% credible intervals (CIs)). However, the marginal Bayesian *R* ^2^, which reflects only the variance explained by environmental predictors (fixed effects), was considerably lower (*R* ^2^ = 0.27 (0.01: 0.51)). This indicates that random effects – both non‐phylogenetic (or species‐level) and phylogenetic – accounted for much of the variation. The nonphylogenetic or species‐level effect had a standard deviation of 1.81 (0.06: 5.62), while the phylogenetic effect had a standard deviation of 1.64 (0.50: 4.73), highlighting that trait variation is structured at both the species and phylogenetic levels. Although none of the regression parameters (environmental predictors) deviated from zero (Fig. 5), the direction of the mean effect revealed ecologically meaningful trends. With the narrowest credible interval, VPD showed the most suggestive and consistent effect, with a positive association with deciduousness (*b* = 1.18 (−1.22: 3.62); Fig. 5), indicating a potential role of atmospheric dryness in shaping leaf habit. By contrast, higher precipitation during the growing season was associated with a lower probability of deciduousness (*b* = −1.92 (−4.99: 1.06)), suggesting a tendency toward evergreen strategies in wetter environments, albeit with substantial uncertainty. Similarly, species occurring in more arid environments (lower AI values) tended to be more likely deciduous (*b* = −1.92 (−4.95: 1.32)), consistent with deciduousness as a drought‐avoidance strategy, despite the greater uncertainty in this estimate. Both soil texture variables also showed negative mean effects on the probability of deciduousness, with sand content (*b* = −1.44 (−4.20: 1.54)) and clay content (*b* = −0.74 (−3.73: 2.19)) exhibiting wide credible intervals. Accordingly, BART partial dependence plots indicate that deciduousness is most strongly and consistently associated with nonlinear gradients of atmospheric dryness (VPD) and aridity (AI), while soil variables exert weaker and more uncertain influences (Fig. S5).

Comparisons of Bayesian *R* ^2^ across model specifications indicated that the baseline model including both AI and GSP explained slightly more variation than the reduced models (Table S4). The reduced models excluding either AI or GSP yielded slightly lower marginal Bayesian *R* ^2^ values (0.22 (< 0.01: 0.49); Table S4) in both cases, whereas the baseline – full – model exhibited a marginal Bayesian *R* ^2^ of 0.27, as reported previously. By contrast, conditional Bayesian *R* ^2^ values were nearly identical across models (*c*. 0.80; Table S4), indicating that most of the explained variance was captured by species‐level and phylogenetic random effects. Retaining both predictors therefore slightly increased the proportion of variation in leaf habit explained by the fixed effects. Consistent with this, BART analyses revealed distinct and nonlinear response patterns for AI and GSP (Fig. S6), indicating that both variables contribute unique information to explaining deciduousness. Specifically, AI was more influential under highly arid conditions, whereas GSP had stronger effects along wetter gradients (Fig. S6). Thus, despite their observed collinearity, the ecological signal of each variable is retained in the full model. Finally, including growth‐form as a fixed effect provided additional ecological context for interpreting variation in leaf habit across growth‐form categories, but only marginally increased the variance explained by the model (conditional Bayesian *R* ^2^ = 0.81 vs 0.80 for the baseline model) and did not increase the variance explained by the fixed effects (marginal Bayesian *R* ^2^ = 0.21 vs 0.27; Table S5).

## Discussion

By integrating evolutionary and environmental perspectives, our study presents a comprehensive assessment of how phylogenetic history and environmental factors jointly influence dry‐season deciduousness in a predominantly tropical group. This approach advances our understanding of drought‐related strategies and their spatial distribution in tropical ecosystems. Deciduous leaf habit is predominant in deserts and dry shrublands, but it also accounts for over 50% of leaf habit variation in temperate biomes with freezing winters (Fig. 2). We found that transitions from evergreen to deciduous were more frequent before 7 Ma, whereas transition rates from deciduous to evergreen remained relatively constant through time. Consequently, after 7 Ma, the relative importance of transitions toward evergreen leaf habit increased, together with higher speciation rates of evergreen lineages, coinciding with the expansion of Cerrado savannas in South America, where evergreen *Mimosa* species predominate. Because the Cerrado occurs mostly on dystrophic, acidic, and nutrient‐poor soils (Furley & Ratter, 1988; Lira‐Martins *et al*., 2022), retaining leaves – and avoiding the nutrient costs of seasonal leaf turnover – may outweigh the advantages of deciduousness, even under a markedly dry season, as also observed in nutrient‐poor Australian savannas where evergreen species predominate (Bowman & Prior, 2005). By contrast, the expansion of succulent biomes, over more fertile soils (Pennington *et al*., 2009), *c*. 15 Ma did not appear to drive shifts in diversification regimes within *Mimosa*. We also found that deciduous species exhibited faster evolutionary rates in niche space characterized by higher VPD than their evergreen counterparts, indicating that deciduous lineages can respond rapidly to variation in atmospheric dryness, as evidenced for environmental niche space for VPD. However, deciduous and evergreen species showed similar responses to soil sand content, suggesting environmental niche convergence or shared environmental constraints. While most of the variation in leaf habit was attributed to nonphylogenetic species‐level factors, higher VPD was associated with an increased probability of deciduousness. These findings highlight that, although deciduous lineages respond rapidly to atmospheric dryness, deciduousness is primarily shaped by the interplay among species identity, evolutionary history, and variation in species‐level traits, such as growth‐form, rather than by environmental conditions alone.

Over the evolutionary history of *Mimosa*, the early expansion of the succulent biome *c*. 15 Ma does not appear to have influenced the transition rates between leaf habits. By contrast, we found a clear temporal shift in the evolutionary role of leaf habit *c*. 7 Ma. Contrary to our initial expectations, the late‐stage success and diversification of evergreen *Mimosa* lineages – evidenced by their higher speciation rates and a sharp decline in transition rates toward deciduousness after 7 Ma (Fig. 3) – may be linked to their radiation into the Cerrado biome. These findings suggest that the ecological dominance of evergreen (Fig. 2c) *Mimosa* is a more recent phenomenon, given that transitions from evergreen to deciduous leaf habits were more frequent before 7 Ma. The timing of this shift coincides with major palaeoenvironmental changes in central South America, including the expansion of fire‐prone savannas, particularly the Cerrado biome (Simon *et al*., 2009). The Cerrado expansion occurred mostly over dystrophic, acidic, and nutrient‐poor soils with low effective CEC (Furley & Ratter, 1988; Lira‐Martins *et al*., 2022). These environmental constraints – especially nutrient‐poor soils and frequent fires – likely limited the persistence of deciduous lineages. By contrast, the earlier expansion of the succulent biome over more fertile and nonacidic soils (Silva de Miranda *et al*., 2018; Lira‐Martins *et al*., 2022) may have imposed less selective pressure against deciduousness. Indeed, across geographic space, soil fertility has been proposed as a key factor underlying the contrasting gradients of deciduousness between savannas and succulent biomes, which often co‐occur under the same climatic envelope in South America (Silva de Miranda *et al*., 2018; Oliveira *et al*., 2021). Our findings extend this pattern to the evolutionary timescale, highlighting the long‐term influence of changes in soil fertility patterns on the evolution of leaf habit.

The severe nutrient limitation likely acts as a strong environmental filter for Cerrado species, favoring species with traits that enhance nutrient‐use efficiency, such as long‐lived leaves (Abrahão *et al*., 2019; Lira‐Martins *et al*., 2022). This is especially true in the *Cerrado rupestre* – a habitat type of the Cerrado with even more nutrient‐impoverished soils and the center of *Mimosa* diversification – where nutrient efficiency is essential for survival and reproduction (Simon & Proença, 2000; Abrahão *et al*., 2019). Deciduousness, which involves high nutrient costs due to a short leaf lifespan, may be disadvantageous under nutrient‐poor conditions (Wright *et al*., 2004), potentially explaining the tendency of deciduous species to occur in more nutrient‐rich environments across geographic space (Cavender‐Bares *et al*., 2004). By contrast, evergreen species avoid these costs by retaining their leaves year‐round; their longer leaf lifespan may help explain their greater success and increased diversification in nutrient‐limited habitats, such as savannas (Bowman & Prior, 2005). Fire is also a strong environmental filter in savannas, potentially shaping the distribution of deciduous and evergreen species across space and time (Givnish, 2002; Bowman & Prior, 2005). Investment in belowground organs is a key driver of diversification in seasonally dry tropical ecosystems of the Americas (Cássia‐Silva *et al*., 2022), especially within *Mimosa*, as it enhances vegetative recovery following fire‐induced structural damage (Simon *et al*., 2009). By remaining largely underground during the dry season, *Mimosa* species may also avoid the costs of deciduousness, which may help explain the prevalence of evergreen *Mimosa* in the Cerrado (savanna) biome (Fig. 2c; M. F. Simon, pers. comm.).

Our study provides macroscale evidence of a direct relationship between atmospheric dryness and dry‐season deciduousness across spatial and evolutionary dimensions in a predominantly tropical lineage. Deciduous lineages can tolerate a broader range of VPD, highlighting VPD as a key environmental predictor of leaf habit variation, even though dry‐season deciduousness primarily decouples plants from atmospheric water demand during the dry season (Oliveira *et al*., 2021). Additionally, species occurring in more arid environments (i.e. those with lower aridity index values) showed a tendency toward deciduousness, while species inhabiting areas with higher precipitation during the GSP were more likely to retain evergreen leaves. Although there is some uncertainty, moderate in the case of VPD and more substantial for the AI and GSP (Fig. 5), these results support our hypothesis of deciduousness as a drought‐avoidance strategy in *Mimosa*. Over evolutionary timescales, deciduous lineages exhibited faster evolutionary responses in VPD niche space (Fig. 4a), suggesting greater environmental niche lability. By contrast, evergreen species appear to have more conserved VPD niches, evolving more slowly in response to changes in atmospheric dryness. Given the accelerating pace of climate change, a more labile environmental niche could confer adaptive advantages, potentially explaining shifts in species composition and functional traits observed in both tropical and high‐latitude ecosystems (Van Der Sande *et al*., 2016; Hisano *et al*., 2021). Transitions in leaf habit, such as from evergreen to deciduous, can profoundly affect ecosystem processes, including carbon cycling (Augusto *et al*., 2015), and may even contribute to the emergence of alternative biome states in tropical regions (Cure *et al*., 2023). However, while VPD appears to be a relatively important predictor of leaf habit and may improve the predictive power of vegetation dynamic models, the influence of soil water availability on dry‐season deciduousness remains less clear.

The role of soil in shaping leaf habit variation in *Mimosa* appears to be more complex and nuanced than initially expected. In our environmental models, soil texture variables showed only weak associations with leaf habit. Clay content was weakly linked to a decreased probability of deciduousness, while sand content was slightly associated with an evergreen leaf habit. The effect of clay content was weak but estimated with relatively higher precision, while the effect of sand content was more negative in magnitude but less precisely estimated. Over evolutionary timescales, however, the best‐supported model for soil sand content revealed no differences between leaf habit regimes, suggesting niche convergence or similar selective pressures acting on both deciduous and evergreen *Mimosa* lineages. These results highlight the potential confounding effects of water and nutrient availability embedded in soil texture. Water limitation generally favors deciduousness, whereas nutrient limitation often favors an evergreen leaf habit. Sandy soils, which are nutrient‐poor due to rapid drainage and low water‐holding capacity, may simultaneously impose both constraints – limiting water access during dry seasons while restricting nutrient retention. As a result, soil conditions may exert opposing selective pressures: favoring deciduousness to avoid seasonal drought but favoring evergreen species to increase nutrient‐use efficiency. This dual selection may help explain the absence of clear associations between soil texture and leaf habit in *Mimosa*, at least with currently available soil texture data. Moreover, the temporal distribution of deciduousness may also have been shaped by major environmental shifts across palaeoecological timescales, as suggested by the diversification model. Historical heterogeneity in both climate and soil conditions that may have influenced the evolution of deciduousness was not captured by our current analyses and warrants further investigation. Future studies should also incorporate more direct proxies of soil nutrient availability – such as soil pH – since soil texture alone may inadequately represent variation in soil fertility.

In sum, variation in leaf habit among *Mimosa* species is best explained by the combined effects of species‐level trait variation and phylogenetic structure. Species‐level effects highlight the interplay between local species adaptations, such as variation in growth form, and environmental conditions in shaping trait variation. Phylogenetic effects, in turn, underscore the role of evolutionary history, suggesting that the physiological mechanisms underlying deciduousness may be conserved over evolutionary timescales. Consequently, dry‐season deciduousness appears to be primarily shaped by the interplay between species identity and shared ancestry rather than by environmental conditions alone. Taken together and considering deciduousness as an important modulator of ecosystem functioning, these findings highlight the critical role of phylogenetic composition and species‐level trait variation in shaping ecosystem processes.

### Conclusions

Our findings reveal a temporal shift in the evolutionary relevance of leaf habit within *Mimosa*, where transitions toward deciduousness played a more prominent role before 7 Ma (Fig. 3), during the early stage of the genus radiation. By contrast, the ecological dominance and diversification of evergreen *Mimosa* species appear to be a more recent phenomenon, taking place in the late stage of *Mimosa* evolutionary history, after 7 Ma, and potentially linked to the emergence of novel environmental constraints, particularly associated with the expansion of the Cerrado in tropical South America. Over evolutionary time, deciduous *Mimosa* species exhibited faster evolutionary responses to changing VPD conditions (Fig. 4), with VPD emerging as the most influential environmental driver of deciduousness over space (Fig. 5). Soil texture variables, such as clay and sand content, showed negative but more uncertain relationships. The complex association between soil and leaf habit suggests that selection may drive contrasting phenomena simultaneously: Low water availability may favor deciduousness, whereas nutrient‐poor soils may favor evergreen leaf habit. As a result, both leaf habits can coexist over time and space, making their ecological drivers difficult to disentangle. By integrating environmental predictors with evolutionary dynamics, our study provides a unified framework for a deeper understanding of the evolutionary and ecological significance of drought‐deciduousness. As comprehensive studies of well‐sampled clades can shed light on broader patterns in plant ecology and evolution, along with the integrative role of deciduousness from individual physiology to ecosystem‐level processes (Vico *et al*., 2017; Donoghue & Edwards, 2019), our findings advance the conceptual understanding of how leaf habit strategies both shape and are shaped by the interplay between evolutionary history, species‐level variation and environmental factors over time. Greater understanding of past evolutionary responses to environmental change can ultimately inform predictions of ecosystem responses under ongoing and future climate change scenarios.

## Competing interests

None declared.

## Author contributions

CCS and RSO conceptualized the study. CCS and JPL developed the methodological approach and conducted the formal analyses, with input from VMS. MFS and EG curated the legume dataset. LHA performed the SSE models using a time‐stratified approach. JCB, RSO, and JPL provided intellectual contributions to the project and assisted with manuscript writing. All co‐authors contributed to writing, reviewing, and revising the manuscript.

## Disclaimer

The New Phytologist Foundation remains neutral with regard to jurisdictional claims in maps and in any institutional affiliations.

## Supporting information


**Dataset S1** *Mimosa* dataset: leaf habit, primary biome classification and environmental variables for all species of *Mimosa* used in the analysis.

**Fig. S1** Prior probability check showing consistency between observed data and replicated posterior predictions.
**Fig. S2** Posterior predictive checks.
**Fig. S3** Empirical cumulative distribution functions (ECDFs) of the posterior distribution of the VPD regression coefficient (b_VPD) under different prior power‐scaling specifications.
**Fig. S4** Empirical cumulative distribution functions (ECDFs) of the posterior distribution of the model intercept (b_Intercept) under different prior power‐scaling specifications.
**Fig. S5** Partial dependence plots from Bayesian Additive Regression Trees (BART) illustrating the marginal effects of all environmental predictors – climatic and soil variables – on deciduousness.
**Fig. S6** Partial dependence plots from Bayesian Additive Regression Trees (BART) showing the marginal effects of (A) aridity index and (B) growing‐season precipitation on the predicted probability of deciduousness in *Mimosa*.
**Notes S1** Bayesian phylogenetic multilevel model.
**Table S1** Model comparison and parameter estimates for alternative speciation and extinction models assessing the dependence of diversification on a binary trait—leaf habit (deciduous = 1, evergreen = 0). We evaluated models incorporating time‐sliced diversification regimes, indicated as ‘change in regime’ in the table (at 7 or 15 million years ago (Ma)), to test how the influence of a binary trait on diversification may vary through time.
**Table S2** Comparative fit of 10 alternative environmental niche evolution models applied to five environmental variables: VPD (vapor pressure deficit), GSP (growing season precipitation, i.e., total precipitation on all growing season days), AI (aridity index), and soil texture fractions—sand (sand_cont) and clay (clay_cont) content.
**Table S3** Prior sensitivity analysis of fixed effects.
**Table S4** Comparison of parameter estimates between the baseline model and reduced models excluding either the aridity index (AI) or growing‐season precipitation (GSP).
**Table S5** Parameter estimates from the baseline Bayesian phylogenetic multilevel model.
Please note: Wiley is not responsible for the content or functionality of any Supporting Information supplied by the authors. Any queries (other than missing material) should be directed to the *New Phytologist* Central Office.

## Data Availability

The *Mimosa* phylogeny used in this paper is publicly available in Vasconcelos *et al*. (2020). The *Mimosa* leaf habit, biome, and environmental data used for the analyses are publicly available as Supporting Information (Dataset S1). All code necessary to conduct the analyses is available at https://doi.org/10.5281/zenodo.17526109.
