# Supplementary material for: Evolutionary and environmental drivers of dry‐season deciduousness in a legume genus
Source: New Phytol. 2026 Apr 11;250(6):3631–45. doi: 10.1111/nph.71148 (PMC13193478; doi:10.1111/nph.71148)
Supplement: Supplementary file 2 — Fig. S1 Prior probability check showing consistency between observed data and replicated posterior predictions. Fig. S2 Posterior predictive checks. Fig. S3 Empirical cumulative distribution functions (ECDFs) of the posterior distribution of the VPD regression coefficient (b_VPD) under different prior power‐scaling specifications. Fig. S4 Empirical cumulative distribution functions (ECDFs) of the posterior distribution of the model intercept (b_Intercept) under different prior power‐scaling specifications. Fig. S5 Partial dependence plots from Bayesian Additive Regression Trees (BART) illustrating the marginal effects of all environmental predictors – climatic and soil variables – on deciduousness. Fig. S6 Partial dependence plots from Bayesian Additive Regression Trees (BART) showing the marginal effects of (A) aridity index and (B) growing‐season precipitation on the predicted probability of deciduousness in Mimosa. Notes S1 Bayesian phylogenetic multilevel model. Table S1 Model comparison and parameter estimates for alternative speciation and extinction models assessing the dependence of diversification on a binary trait—leaf habit (deciduous = 1, evergreen = 0). We evaluated models incorporating time‐sliced diversification regimes, indicated as ‘change in regime’ in the table (at 7 or 15 million years ago (Ma)), to test how the influence of a binary trait on diversification may vary through time. Table S2 Comparative fit of 10 alternative environmental niche evolution models applied to five environmental variables: VPD (vapor pressure deficit), GSP (growing season precipitation, i.e., total precipitation on all growing season days), AI (aridity index), and soil texture fractions—sand (sand_cont) and clay (clay_cont) content. Table S3 Prior sensitivity analysis of fixed effects. Table S4 Comparison of parameter estimates between the baseline model and reduced models excluding either the aridity index (AI) or growing‐season precipitation (GSP). Table S5 Parameter [file NPH-250-3631-s002.docx]

**New Phytologist Supporting Information**

**Article title:** Evolutionary and environmental drivers of dry-season deciduousness in a legume genus

**Authors:** Cibele Cássia-Silva, Jeannine Cavender-Bares, Marcelo F. Simon, Leonel Herrera-Alsina, Vinicius Marcilio-Silva, Edeline Gagnon, Rafael Silva Oliveira, Jesús N. Pinto-Ledezma

**Article acceptance date**: 16 March 2026

**Dataset S1:** *Mimosa* metadata: leaf habit, primary biome classification and environmental variables for all species of *Mimosa* used in the analysis.

**Note S1**: Bayesian phylogenetic multilevel model implemented in Stan using the “brms” R package (Bürkner, 2017).

*y_i_*=*α+β_i_K*+(1∣gr(phy,cov=phylo_VCV))+(1∣Species)

where:

- *y_i_* is the response variable (leaf habit) for species *i*.
- *α* is the model intercept.
- *β_i_K* represents the effects of all environmental predictors *K*: Aridity Index (AI), Vapor Pressure Deficit (VPD), Growing Season Precipitation (GSP), and soil texture fractions (clay and sand).
- (1 ∣ gr (phy,cov=phylo_VCV)) is a **phylogenetic is a phylogenetic grouping-level,** which models the expected correlation among species due to shared evolutionary history. Here, *phy* refers to the *Mimosa* phylogenetic tree, and *phylo_VCV* is the variance-covariance matrix derived from the tree using the “ape” R package (Paradis *et al.*, 2004) and standardized to a correlation matrix.
- (1∣Species) is an additional species group-level capturing variation not explained by phylogeny.

Note that this model structure allows variance to be partitioned into phylogenetic and non-phylogenetic components, while simultaneously estimating the effects of environmental predictors on leaf habit.

**Figure S1***:* Prior probability check showing consistency between observed data and replicated posterior predictions. (**A**) Prior predictive check helps us to diagnose if the data is consistent with the prior’s specification. As the generated data (thin light blue lines) do not accurately resemble the observed data (black line), we can conclude that the priors are moderately informative. (**B**) Probability density functions for the slope (b) of the VPD variable (light blue color) overlapped with the used prior N (mean = 0, SD = 2) (gray color).

*
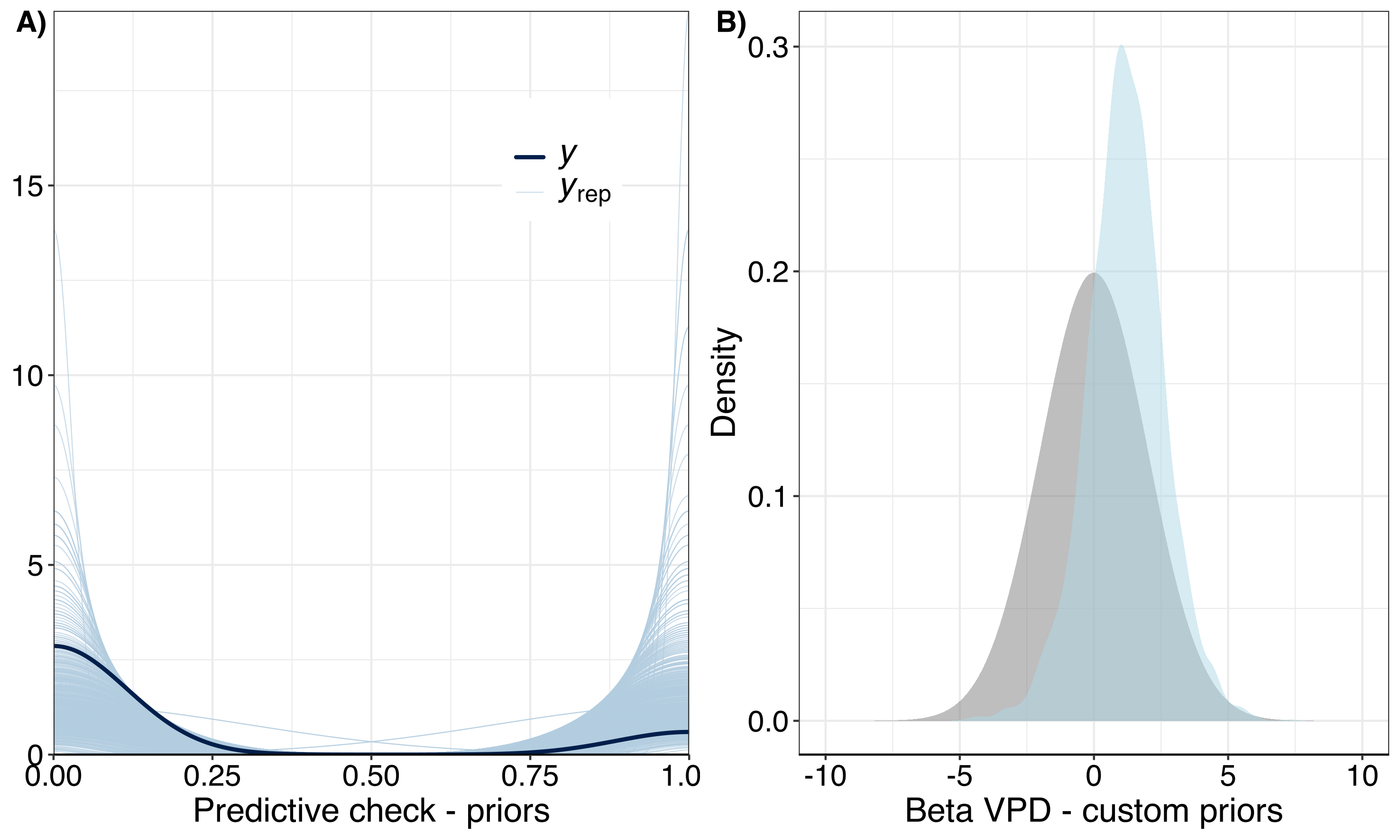
*

**Table S1**: Model comparison and parameter estimates for alternative speciation and extinction models assessing the dependence of diversification on a binary trait—leaf habit (deciduous = 1, evergreen = 0). We evaluated models incorporating time-sliced diversification regimes, indicated as “**change in regime”** in the table (at 7 or 15 million years ago [Ma]), to test how the influence of a binary trait on diversification may vary through time. Four main time-structured scenarios were tested:

1. Dependent diversification early (**Early history** in the table) + independent diversification late (**Late history** in the table),
2. Independent early + dependent late,
3. Dependent early + dependent late, and
4. Independent early + independent late.

For each scenario, we compared models assuming either symmetric transitions between leaf habit states (i.e., equal transition rates between deciduous and evergreen) or asymmetric transitions, indicated as “**transition mode**” in the table. We also compared models with either time-constant or time-variable transition rates (i.e., allowing distinct rates before and after the specified time point), indicated as “**transition rates over time**” in the table. Model fit was assessed using weighted Akaike information criterion (AIC weight). The best-supported model (i.e., the one with the highest AIC weight) is highlighted in bold.

| **Early history** | **Late history** | **Transition mode** | **Transition rates over time** | **Change in regime (Ma)** | **Log-likelihoods** | **No. free parameters** | **AIC weights** |
| --- | --- | --- | --- | --- | --- | --- | --- |
| **Independent** | **Dependent** | **Asymmetrical** | **Time-variable** | **7** | **-56.979** | **7** | **0.9976** |
| Independent | Dependent | Asymmetrical | Time-constant | 7 | -65.211 | 5 | 0.0020 |
| Independent | Dependent | Symmetrical | Time-variable | 7 | -66.834 | 5 | 0.0004 |
| Independent | Dependent | Symmetrical | Time-constant | 7 | -70.483 | 4 | < 0.00001 |
| Independent | Dependent | Asymmetrical | Time-constant | 15 | -111.555 | 5 | < 0.00001 |
| Independent | Dependent | Asymmetrical | Time-variable | 15 | -109.708 | 7 | < 0.00001 |
| Independent | Dependent | Symmetrical | Time-variable | 15 | -118.876 | 5 | < 0.00001 |
| Independent | Independent | Symmetrical | Time-constant | 15 | -827.617 | 4 | < 0.00001 |
| Independent | Independent | Symmetrical | Time-constant | 7 | -827.617 | 4 | < 0.00001 |
| Independent | Independent | Symmetrical | Time-variable | 15 | -826.773 | 5 | < 0.00001 |
| Independent | Independent | Symmetrical | Time-variable | 7 | -824.963 | 5 | < 0.00001 |
| Independent | Independent | Asymmetrical | Time-constant | 15 | -818.608 | 5 | < 0.00001 |
| Independent | Independent | Asymmetrical | Time-constant | 7 | -818.608 | 5 | < 0.00001 |
| Independent | Independent | Asymmetrical | Time-variable | 15 | -818.243 | 7 | < 0.00001 |
| Independent | Independent | Asymmetrical | Time-variable | 7 | -816.113 | 7 | < 0.00001 |
| Independent | Dependent | Symmetrical | Time-constant | 15 | -827.915 | 4 | < 0.00001 |
| Dependent | Independent | Symmetrical | Time-constant | 15 | -841.800 | 4 | < 0.00001 |
| Dependent | Independent | Symmetrical | Time-constant | 7 | -835.310 | 4 | < 0.00001 |
| Dependent | Independent | Symmetrical | Time-variable | 15 | -841.036 | 5 | < 0.00001 |
| Dependent | Independent | Symmetrical | Time-variable | 7 | -832.896 | 5 | < 0.00001 |
| Dependent | Independent | Asymmetrical | Time-constant | 15 | -832.446 | 5 | < 0.00001 |
| Dependent | Independent | Asymmetrical | Time-constant | 7 | -828.593 | 5 | < 0.00001 |
| Dependent | Independent | Asymmetrical | Time-variable | 15 | -832.302 | 7 | < 0.00001 |
| Dependent | Independent | Asymmetrical | Time-variable | 7 | -827.126 | 7 | < 0.00001 |
| Dependent | Dependent | Symmetrical | Time-constant | 15 | -841.542 | 4 | < 0.00001 |
| Dependent | Dependent | Symmetrical | Time-constant | 7 | -841.542 | 4 | < 0.00001 |
| Dependent | Dependent | Symmetrical | Time-variable | 15 | -841.008 | 5 | < 0.00001 |
| Dependent | Dependent | Symmetrical | Time-variable | 7 | -840.575 | 5 | < 0.00001 |
| Dependent | Dependent | Asymmetrical | Time-constant | 15 | -833.670 | 5 | < 0.00001 |
| Dependent | Dependent | Asymmetrical | Time-constant | 7 | -833.670 | 5 | < 0.00001 |
| Dependent | Dependent | Asymmetrical | Time-variable | 15.1 | -834.863 | 7 | < 0.00001 |
| Dependent | Dependent | Asymmetrical | Time-variable | 7 | -832.380 | 7 | < 0.00001 |

**Table S2:** Comparative fit of ten alternative environmental niche evolution models applied to five environmental variables: **VPD** (vapor pressure deficit), **GSP** (growing season precipitation, i.e., total precipitation on all growing season days), **AI** (aridity index), and soil texture fractions—sand (**sand_cont**) and clay (**clay_cont**) content. Models were fit for deciduous and evergreen Mimosa species using the hOUwie framework (Boyko *et al.*, 2023), implemented via the R package **OUwie** (Beaulieu *et al.*, 2012). Model descriptions are provided in Table 2 of the main text. Model fit was assessed using the weighted Akaike information criterion (**AIC weight**). The best-supported model for each environmental variable (i.e., the one with the highest AIC weight) is highlighted in bold and in the graph below.

| **Model** | **VPD** | **GSP** | **AI** | **Sand_cont** | **Clay_cont** |
| --- | --- | --- | --- | --- | --- |
| **BM1** | 5.8e-11 | 1.13e-04 | 2.81e-03 | 1.70e-17 | 9.15e-20 |
| **BMV** | 1.96e-11 | 4.11e-05 | 1.12e-03 | 5.19e-18 | 2.79e-20 |
| **OU1** | 1.76e-02 | 2.22e-01 | 2.18e-06 | **6.50e-01** | **3.68e-01** |
| **OUA** | 8.26e-03 | **3.75e-01** | 4.16e-04 | 1.95e-18 | 9.92e-21 |
| **OUV** | **9.46e-01** | 2.93e-01 | **4.27e-01** | 1.10e-01 | 2.23e-01 |
| **OUM** | 2.35e-02 | 5.80e-02 | 2.97e-01 | 1.77e-01 | 3.29e-01 |
| **OUVA** | 2.22e-03 | 4.79e-06 | 1.28e-04 | 6.40e-19 | 3.51e-21 |
| **OUMV** | 2.37e-03 | 3.28e-04 | 1.25e-01 | 4.80e-19 | 8.00e-02 |
| **OUMA** | — | 1.58e-02 | 1.23e-01 | 6.24e-02 | 2.92e-27 |
| **OUMVA** | 6.69e-13 | 3.62e-02 | 2.44e-02 | 2.02e-19 | — |

**Figure S2**: Posterior predictive checks. (**A**) Kernel density estimate of the observed data (black line) and simulated data based on our model (thin light blue lines). As the model-generated data resemble the observed data, our model presents a good predictive performance. (**B to D**) Posterior predictive checks for different statistical moments.


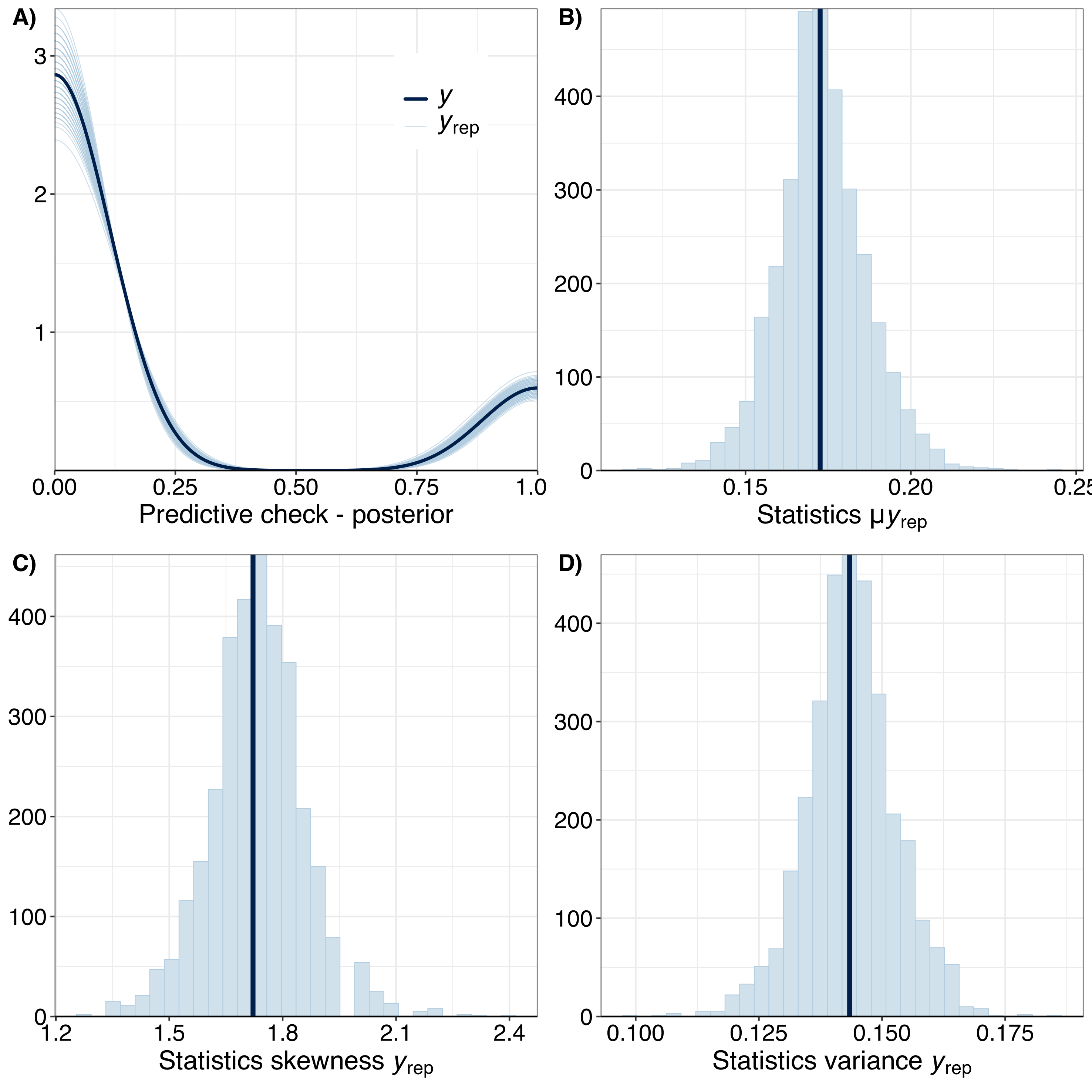


**Table S3. Prior sensitivity analysis of fixed effects.** Posterior median estimates and 95% credible intervals for fixed effects under alternative prior specifications [original prior (prior used in the baseline phylogenetic multilevel model), wide prior, and wide prior v2]. Estimates are shown on the logit scale. A model fitted with fully *flat priors* showed poor convergence and implausible parameter magnitudes and was therefore excluded from this comparative table.

| **Predictor** | **Original prior** | **Wide prior** | **Wide prior v2** |
| --- | --- | --- | --- |
| **Intercept** | −2.46 [−8.27, 2.15] | −2.58 [−11.06, 3.06] | −2.79 [−9.54, 2.90] |
| **VPD** | 1.18 [−1.22, 3.62] | 2.39 [−1.66, 7.27] | 1.17 [−1.27, 3.59] |
| **Growing-season precipitation (GSP)** | −1.92 [−4.99, 1.06] | −4.10 [−11.33, 2.55] | −1.92 [−5.16, 1.19] |
| **Aridity Index (AI)** | −1.92 [−4.95, 1.32] | −3.79 [−11.56, 3.17] | −1.87 [−5.05, 1.43] |
| **Sand soil content** | −1.44 [−4.20, 1.54] | −4.29 [−10.20, 1.38] | −1.36 [−4.16, 1.80] |

### ****Figure S3.**** Empirical cumulative distribution functions (ECDFs) of the posterior distribution of the VPD regression coefficient (b_VPD) under different prior power-scaling specifications. Curves correspond to power-scaling values α = 0.8, 1.0, and 1.25 applied to the prior and likelihood components. Results are shown for the posterior model and for alternative prior formulations (posterior: original prior, flat, wide, and wide v2). The strong overlap among ECDFs across power-scaling values and prior specifications indicates that posterior estimates of the VPD effect are robust to prior strength and likelihood weighting.

###
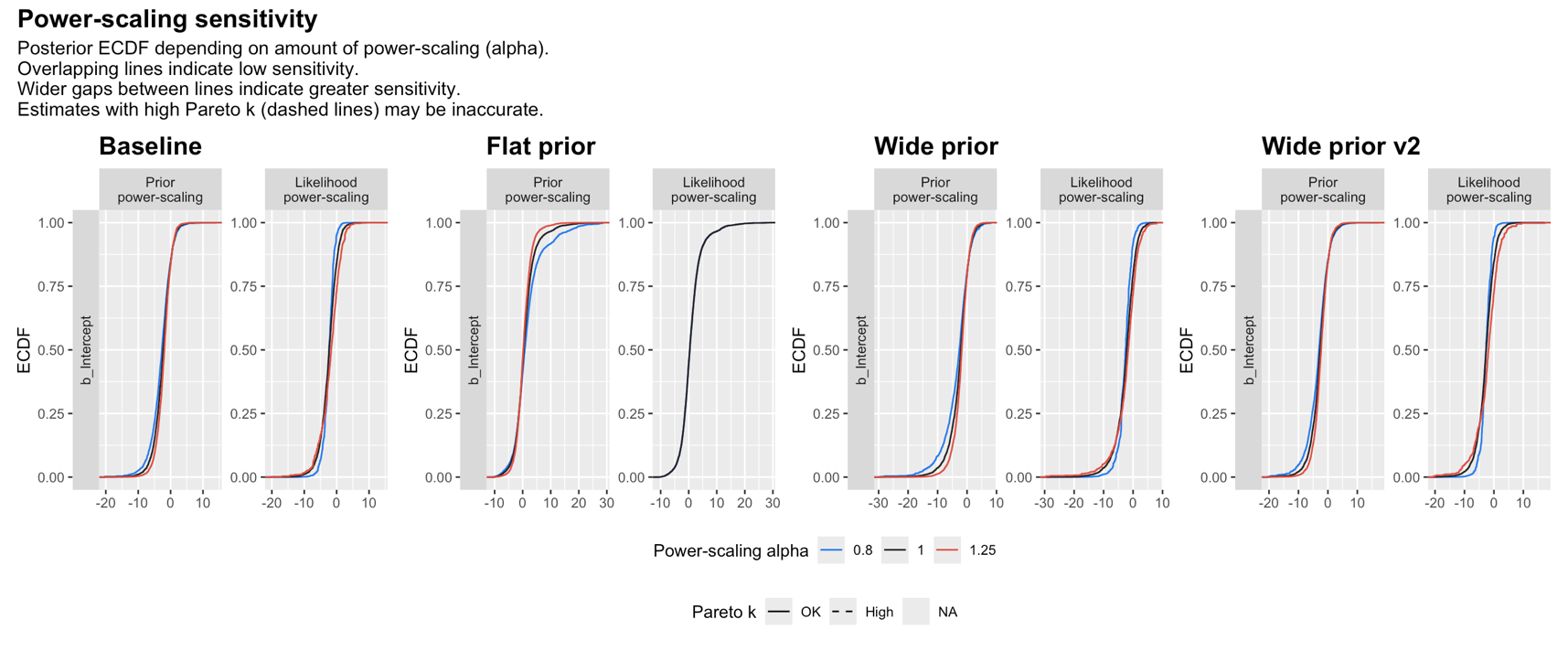

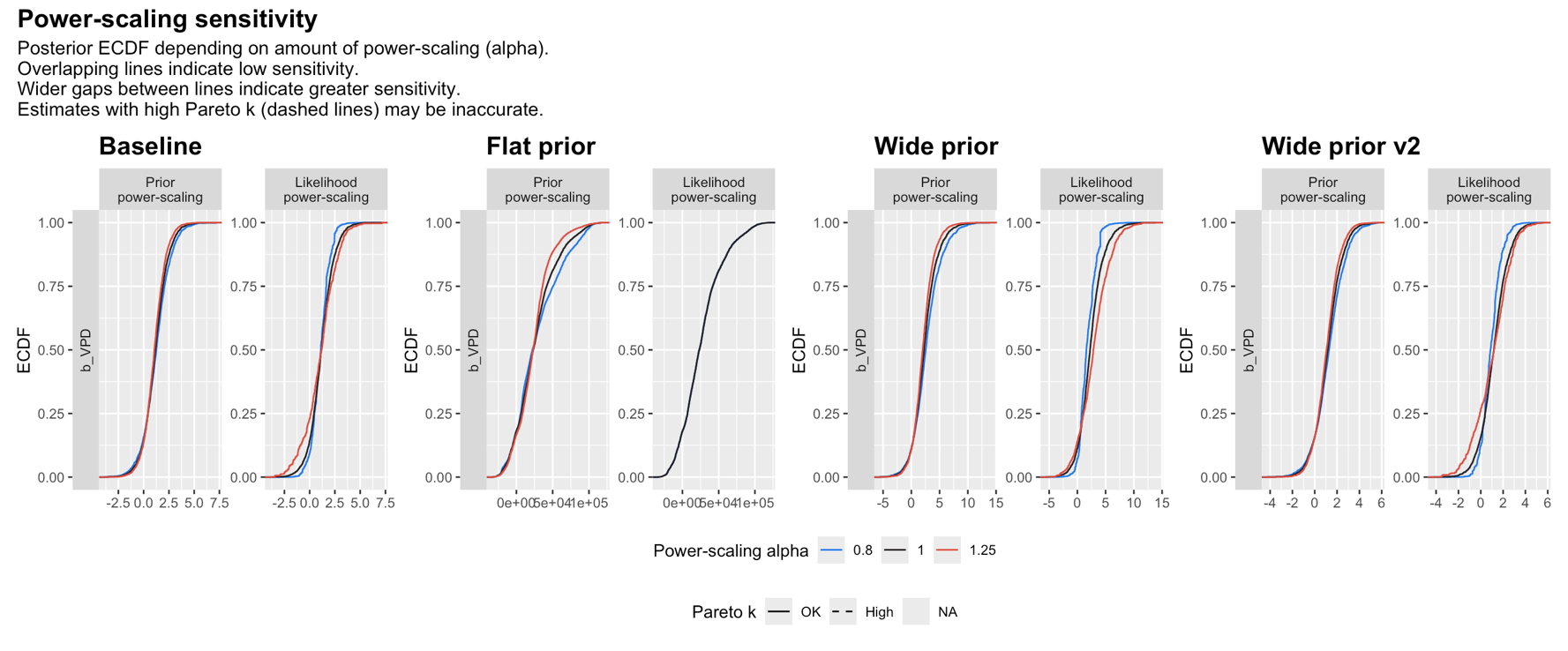
****Figure S4.**** Empirical cumulative distribution functions (ECDFs) of the posterior distribution of the model intercept (b_Intercept) under different prior power-scaling specifications. Curves represent power-scaling values α=0.8, 1.0, and 1.25 applied to the prior and likelihood components across the posterior model and alternative prior formulations (baseline: original prior used on baseline model, flat, wide, and wide v2). The high degree of overlap among ECDFs indicates that intercept estimates are largely insensitive to prior power-scaling choices, supporting the robustness of model inference.

**Table S4:** Comparison of parameter estimates between the baseline model and reduced models excluding either the aridity index (AI) or growing-season precipitation (GSP). Values are posterior means with 95% credible intervals (CIs) in brackets. Random effects are reported as standard deviations (SD). Both models were fitted using a Bernoulli likelihood with a logit link.

| **Component** | **Parameter** | **Baseline model -** **full model** | **no AI** | **no GSP** |
| --- | --- | --- | --- | --- |
| **Fixed effects** | Intercept | −2.46 [−8.27: 2.15] | −2.32 [−7.65: 2.43] | −2.38 [−7.94: 2.22] |
|  | VPD | 1.18 [−1.22: 3.62] | 1.18 [−1.23: 3.57] | 1.14 [−1.21: 3.57] |
|  | Precipitation (GSP) | −1.92 [−4.99: 1.06] | −2.71 [−5.54: 0.13] | − |
|  | Aridity Index | −1.92 [−4.95: 1.32] | − | −2.80 [−5.66: 0.25] |
|  | Soil sand content | −1.44 [−4.20: 1.54] | −1.28 [−4.02: 1.80] | −1.29 [−4.07: 1.73] |
|  | Soil clay content | −0.74 [−3.73: 2.19] | −1.03 [−4.04: 1.97] | −0.79 [3.82: 2.29] |
| **Random effects (SD)** | Species (non-phylogenetic) | 1.81 [0.06: 5.62] | 1.78 [0.07: 5.79] | 1.79 [0.05: 6.06] |
|  | Phylogeny | 1.64 [0.50: 4.73] | 1.78 [0.50: 5.79] | 1.67 [0.48: 5.32] |
| **Model fit** | **Bayesian *R²* (conditional)** | **0.80 [0.64: 0.94]** | **0.80 [0.633: 0.95]** | **0.796 [0.63: 0.95]** |
|  | **Bayesian *R²* (marginal)** | **0.27 [<0.01: 0.51]** | **0.22 [<0.01: 0.48]** | **0.22 [<0.01: 0.49]** |
|  | N | 226 | 226 | 226 |


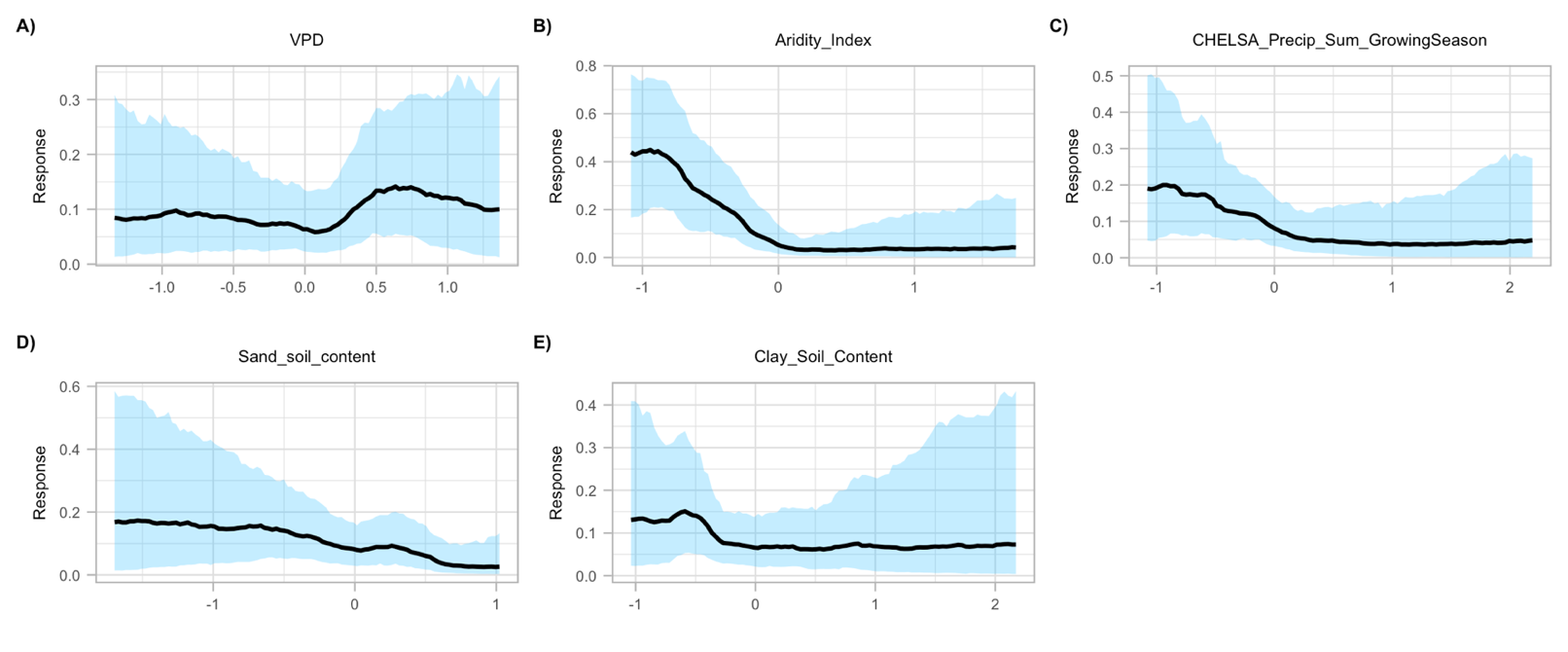
**Figure S5:** Partial dependence plots from Bayesian Additive Regression Trees (BART) illustrating the marginal effects of all environmental predictors—climatic and soil variables—on deciduousness. Responses are shown as posterior means (black lines) with 95% credible intervals (shaded areas). Vapor pressure deficit—VPD (**A**) and Aridity index (**B**) exhibit the strongest nonlinear associations, whereas growing-season precipitation (**C**) and soil texture variables (**D** and **E**) show weaker effects.

**Figure S6:** Partial dependence plots from Bayesian Additive Regression Trees (BART) showing the marginal effects of (**A**) aridity index and (**B**) growing-season precipitation on the predicted probability of deciduousness in *Mimosa.* These two variables were highly collinear (*r* = 0.93). The plots are averaged over all other variables. Black lines represent posterior mean responses, and shaded areas indicate 95% credible intervals.


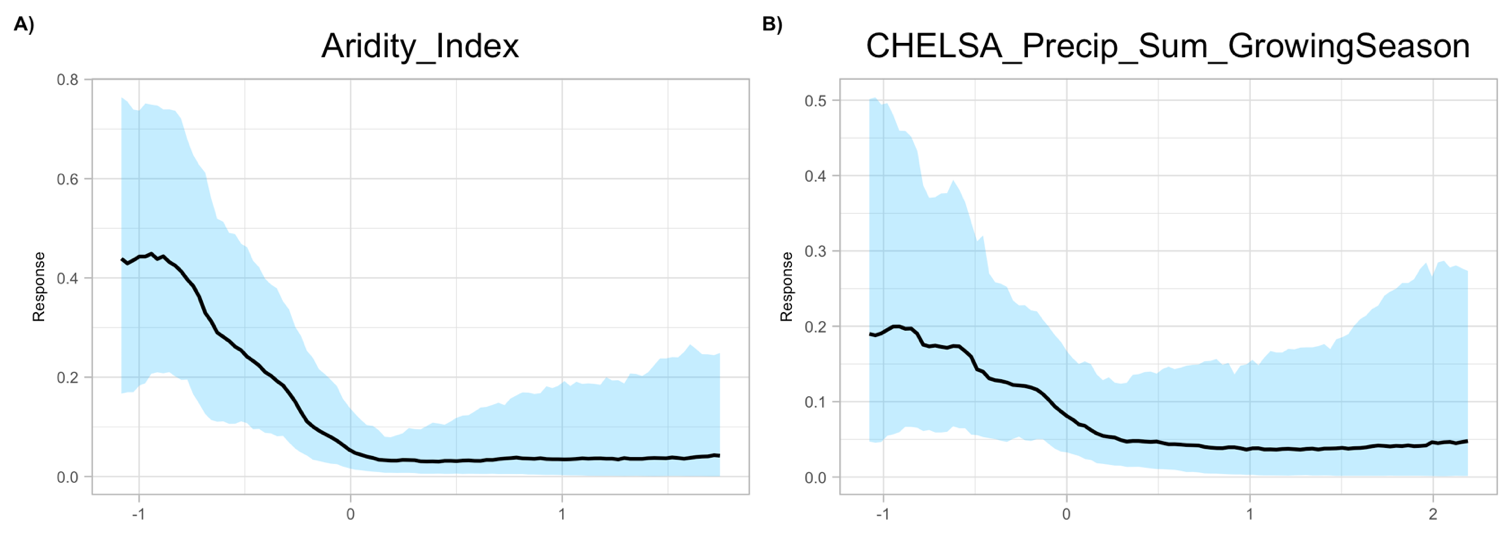


| **Component** | **Parameter** | **Baseline model** | **Growth-form (GF) model** |
| --- | --- | --- | --- |
| **Fixed effects** | Intercept | −2.46 [−8.27: 2.15] | −3.74 [−10.14: 2.84] |
|  | VPD | 1.18 [−1.22: 3.62] | 0.98 [−1.43: 3.67] |
|  | Precipitation (GSP) | −1.92 [−4.99: 1.06] | −2.06 [−5.21: 0.92] |
|  | Aridity Index | −1.92 [−4.95: 1.32] | −2.03 [−5.39: 1.27] |
|  | Soil sand content | −1.44 [−4.20: 1.54] | −1.47 [−4.36: 1.73] |
|  | Soil clay content | −0.74 [−3.73: 2.19] | −0.72 [−3.83: 2.44] |
|  | Growth form | − | 0.89 [−1.34: 3.12] |
| **Random effects (SD)** | Species (non-phylogenetic) | 1.81 [0.06: 5.62] | 2.02 [0.08: 5.93] |
|  | Phylogeny | 1.64 [0.50: 4.73] | 1.72 [0.53: 4.77] |
| **Model fit** | **Bayesian *R²* (conditional)** | **0.80 [0.64: 0.94]** | **0.81 [0.65: 0.95]** |
|  | **Bayesian *R²* (marginal)** | **0.27 [<0.01: 0.51]** | **0.21 [<0.01: 0.5]** |
|  | N | 226 | 226 |

### Table S5: Parameter estimates from the baseline Bayesian phylogenetic multilevel model and the alternative model including growth-form (GF) as a fixed effect. Values are posterior means with 95% credible intervals (CIs) in brackets. Random effects are reported as standard deviations (SD). Both models were fitted using a Bernoulli likelihood with a logit link.

**REFERENCES**

**Beaulieu JM, Jhwueng D-C, Boettiger C, O’Meara BC**. **2012**. Modeling stabilizing selection: expanding the Ornstein-Uhlenbeck model of adaptive evolution: expanding the Ornstein-Uhlenbeck model. *Evolution* **66**: 2369–2383

**Boyko JD, O’Meara BC, Beaulieu JM**. **2023**. A novel method for jointly modeling the evolution of discrete and continuous traits. *Evolution* **77**: 836–851.

**Bürkner P. C**. **2017**. brms: An R Package for Bayesian Multilevel Models Using Stan. *Journal of Statistical Software* **80**: 1-28. doi: [10.18637/jss.v080.i01](https://doi.org/10.18637/jss.v080.i01)

**Paradis E, Claude J, Strimmer K**. **2004**. APE: Analyses of Phylogenetics and Evolution in R language. *Bioinformatics* **20**: 289–290.
